# Phenotypic and Genotypic Characteristics of Mastocytosis According to the Age of Onset

**DOI:** 10.1371/journal.pone.0001906

**Published:** 2008-04-09

**Authors:** Fanny Lanternier, Annick Cohen-Akenine, Fabienne Palmerini, Frédéric Feger, Ying Yang, Yael Zermati, Stéphane Barète, Beatrix Sans, Cédric Baude, David Ghez, Felipe Suarez, Richard Delarue, Philippe Casassus, Christine Bodemer, Adeline Catteau, Frédérique Soppelsa, Katia Hanssens, Michel Arock, Hagay Sobol, Sylvie Fraitag, Danièle Canioni, Alain Moussy, Jean Marie Launay, Patrice Dubreuil, Olivier Hermine, Olivier Lortholary, for the AFIRMM network

**Affiliations:** 1 Université Paris V, Service de maladies infectieuses et tropicales, Centre de référence des mastocytoses, Hôpital Necker Enfants malades, Centre d'Infectiologie Necker-Pasteur, Paris, France; 2 AFIRMM network, Paris, France; 3 Inserm: Centre de Recherches en Cancérologie de Marseille, Hématopoïèse Moléculaire et Fonctionnelle, U599, Marseille, France; 4 Institut Paoli-Calmettes, Département de Biopathologie, Marseille, France; 5 Université Méditerranée, Marseille, France; 6 Laboratoire de Biotechnologies et de Pharmacologie génétique Appliquée CNRS UMR8113 Ecole Normale Supérieure de Cachan, Cachan, France; 7 Service de dermatologie, centre de référence des mastocytoses, Hôpital Tenon, Paris, France; 8 Service de dermatologie, Hôpital Purpan, Toulouse, France; 9 Service d'hématologie, Centre de référence des mastocytoses, Hôpital Necker Enfants malades, Université Paris V, IFR Necker, Paris, France; 10 Service d'hématologie, Hôpital Avicenne, Bobigny, France; 11 Service de dermatologie, Centre de référence des mastocytoses, Hôpital Necker Enfants malades, Université Paris V, IFR Necker, Paris, France; 12 Service d'anatomopathologie, Hôpital Necker Enfants malades, Paris, France; 13 Service de Biochimie, Hôpital Lariboisière, Paris, France; 14 CNRS UMR 8147, Paris, France; University of California Merced, United States of America

## Abstract

Adult's mastocytosis is usually associated with persistent systemic involvement and *c-kit* 816 mutation, while pediatrics disease is mostly limited to the skin and often resolves spontaneously. We prospectively included 142 adult patients with histologically proven mastocytosis. We compared phenotypic and genotypic features of adults patients whose disease started during childhood (Group 1, n = 28) with those of patients whose disease started at adult's age (Group 2, n = 114). Genotypic analysis was performed on skin biopsy by sequencing of *c-kit* exons 17 and 8 to 13. According to WHO classification, the percentage of systemic disease was similar (75 vs. 73%) in 2 groups. *C-kit* 816 mutation was found in 42% and 77% of patients in groups 1 and 2, respectively (p<0.001). 816 *c-kit* mutation was associated with systemic mastocytosis in group 2 (87% of patients with systemic mastocytosis vs. 45% with cutaneous mastocytosis, p = 0.0001). Other *c-kit* activating mutations were found in 23% of patients with mastocytosis' onset before the age of 5, 0% between 6 and 15 years and 2% at adults' age (p<0.001). In conclusion, pathogenesis of mastocytosis significantly differs according to the age of disease's onset. Our data may have major therapeutic relevance when considering c-kit-targeted therapy.

## Introduction

Mastocytosis is a heterogeneous disease characterized by mast cell accumulation in various organs. Some cases are complicated with organ insufficiency and/or symptoms due either to mast cell infiltration or mediators release. Tissues that are commonly involved are skin, bone marrow, gastrointestinal tract, liver and skeletal systems[1]. The majority of mastocytosis cases occur in children (65%)[2], mostly as isolated cutaneous forms while less than 20% are complicated by a systemic dissemination[3]. Most of the cases resolve by puberty[3]. In contrast, patients with mastocytosis starting at the adult's age present more often persistent systemic involvement.

Stem cell factor (SCF) is the major growth factor for mast cell survival and differentiation and interacts with its cognate receptor KIT, a tyrosine kinase encoded by the *c-kit* gene. *C-kit* proto-oncogene activation results in mastocytes accumulation and abnormal migration and activation in various tissues[4], [5]. In neoplastic mast cell lines, valine or tyrosine substitution for an aspartate in *c-kit* codon 816 results in constitutive phosphorylation and activation of KIT[6]. In early reports, Asp816Val was found in peripheral blood of 27% of 55 adult patients with mastocytosis mainly of systemic forms or associated with clonal haematological disorders [7]–[9]. Currently, it is well admitted that this mutation was also reported in skin and bone marrow in the vast majority of adult patients with systemic or cutaneous mastocytosis[7]–[11]. In contrast, mutations in the *c-kit* gene are considered as rare in children[7]–[10]. However, a recent study reports presence of 816 mutations in 11 out of 13 adults with the pediatric onset of cutaneous mastocytosis and in all children with systemic mastocytosis (SM)[12]. Taken together these findings may explain differences in clinical presentation and outcome according to age of onset, and particularly the resolution of the disease in children's mastocytosis. The finding of these mutations may have important implications for pathogenesis understanding, prognosis and therapeutics particularly regarding the potential use of c-kit inhibitors.

To our knowledge, no large study has attempted to describe characteristics of adult's mastocytosis according to the age of disease's onset. In this report, we compared phenotype and *c-kit* genotype in a large prospective cohort of adults' patients with histologically confirmed mastocytosis according to their childhood or adulthood age of onset.

## Results

A hundred and forty-two patients with mastocytosis were recruited, 28 patients in group 1 (13 in group 1A and 15 in group 1B) and 114 in group 2. For the whole population, thirty-five percent were male, with a mean age of 46±14 years at the time of inclusion. Seventy-three percent had histologically confirmed (bone marrow or other histologically proven organ involvement) systemic mastocytosis (SM) with 63% ISM, 6% ASM, 3% SSM and 1% SM-ASHNMD. The most frequently involved organ was bone marrow, concerning 64% of the patients, then skeletal system (40%), gastrointestinal system (12%) and liver (4%). Considering skin lesions, 90% of the patients presented with urticaria pigmentosa, 27% with diffuse cutaneous mastocytosis, and 14% with TEMP. Clinical examination showed hepatomegaly for 12% patients, splenomegaly for 9% and adenomegaly for 7%. The most frequent symptoms were asthenia (65% of the patients), flush (56%), diarrhoea (50%), abdominal pain (47%), skeletal pain (43%), and anaphylactic shock or blackout (17%). Tryptase level was above 20 ng/ml for 65% of the patients.

Comparison of patients' characteristics between groups 1 and 2 is reported in table 1. As expected patients whose disease started in adulthood had a shorter duration of mastocytosis evolution. Patients with childhood onset mastocytosis had less frequent serum tryptase elevation and tended to present more frequent liver involvement. Gender, presence of systemic involvement, type of skin lesion, bone marrow infiltration, gastrointestinal involvement, hepatomegaly, splenomegaly, adenomegaly, weakness, anaphylactoid shock or blackout, nausea, abdominal pain, diarrhea, osteoporosis and prevalence of X-ray bone lesions did not statistically differ between two groups. Hematological and liver biological parameters did not differ according to the age of onset (data not shown). Familial mastocytosis was found in 12 patients (8%).

**Table 1:** Characteristics of 142 adults with mastocytosis according to their age of onset.

Age of onset	Group 1: ≤15 years (n = 28)	Group 2: >15 years (n = 114)	P
	N (%)	N (%)	
Females	19 (68)	74 (65)	0.700
Age	32±10	49±13	<0.0001
Age of onset	7±6	36±13	<0.0001
Duration of mastocytosis evolution (years)	25±13	13±11	<0.0001
WHO classification			
CM	7 (25)	31 (27)	
ISM	17 (60)	72 (63)	
ASM	3 (11)	6 (5)	
SSM	1 (3)	3 (3)	
SM-AHNMD	0 (0)	2 (2)	0.820
SM	21 (75)	83 (73)	0.800
Hepatomegaly	5 (15)	14 (11)	0.524
Splenomegaly	4 (12)	10 (8)	0.459
Adenomegaly	1 (8)	7 (7)	0.890
Clinical cutaneous form			
Urticaria pigmentosa	24 (86)	104 (91)	0.381
Diffuse cutaneous mastocytosis	6 (21)	32 (33)	0.316
TMEP	4 (14)	13 (14)	0.841
Organ involvement			
Gastrointestinal	5 (18)	12 (11)	0.284
Bone marrow	16 (57)	75 (68)	0.414
Liver	3 (11)	3 (3)	0.058
Skeletal involvement	11 (39)	47 (41)	0.839
Systemic symptoms			
Flush	19 (68)	60 (54)	0.188
Weakness	15 (54)	71 (67)	0.188
Anaphylactoid shock	7 (26)	17 (13)	0.200
Abdominal pain	15 (54)	47 (46)	0.456
Diarrhoea	12 (43)	56 (52)	0.396
Nausea	6 (26)	12 (26)	1.000
Skeletal pain	15 (54)	42 (40)	0.184
Elevated tryptase serum levels (>20 ng/ml)	12 (48)	70 (69)	0.045

CM: cutaneous mastocytosis, ISM: indolent systemic mastocytosis, ASM: aggressive systemic mastocytosis, SSM: smouldering systemic matocytosis, SM-AHNMD: systemic mastocytosis with an associated haematological clonal non-mast cell lineage disease, TMPE: telangiectasia macularis eruptive perstans.

Genotypic characteristics of patients are reported in table 2. Sixty eight percent of the patients presented a D816X mutation of *c-kit* gene in the exon number 17. Patients with an adult onset mastocytosis had more frequently D816X *c-kit* mutation (77% in group 2 vs. 42% of patients in group 1, p<0.001). Elevated tryptase serum levels (>20 ng/ml) were more frequently observed in the group of patients with the presence of 816 c-kit mutation (88%) vs those without (44%) (p<0.0001). Presence of D816X *c-kit* mutation was not associated with duration of mastocytosis evolution (16.25±12.25 years in patients without mutation vs. 14±11.65 in patients with the mutation, p = 0.328).

**Table 2:** Exons 8 through 13 and 17 mutations according to mastocytosis age of onset.

Age of onset	WT	Other mutations	Mut 816
Group 1A (n = 12)	3 (25)	3 (25)	6 (50)
Group 1B (n = 14)	9 (64)	0 (0)	5 (36)
Group 2 (n = 112)	24 (21)	2 (2)	86 (77)

Data are expressed in number (percentage), 816: substitution of valine in 816 codon of *c-kit*, Other mutations: mutation in exons 8 through 13. WT: absence of mutation in exons 8 through 13 and 17. Complete genotype was not available for 4 816 WT patients; 1 patient in group 1A, 1 in group 1B and 2 in group 2.

Mutation screening from exons 8 through 13 was performed for 816 wild type patients except 1 patient in group 1A, 1 patient in group 1B and 2 in group 2 because the amount of RNA was not sufficient and a new biopsy was denied. Non-816 mutations were mostly found in group 1A (25% in group 1A vs. 0% in group 1B vs. 2% in group 2, p = 0.002). In group 1A, 2 patients presented with Del419D deletion in exon 8 and 1 with K509I mutation in exon 9. In group 2, 1 patient presented Del419D deletion in exon 8 and 1 patient with V560G mutation in exon 11. We report the main characteristics of these patients with unusual *c-kit* mutations in table 3.

**Table 3:** Description of 5 adult patients with mastocytosis not related to 816 c-kit mutation.

Mutation	Exon	Gender	Age of onset	WHO classification	Familial form
K509I	9	M	<1 year	CM	No
Del419D	8	M	<1 year	ISM (bone marrow)	No
Del419D	8	F	5 years	ISM (bone marrow)	No
Del419D	8	F	41 years	ISM (bone marrow, gastrointestinal)	No
V560G	11	F	53 years	CM	No

We studied the association between genotype and phenotype in groups 1 and 2, respectively (table 4). No association between genotype and mastocytosis form according to the WHO classification were evidenced in group 1, 71% vs 57% were mutated for 816*ckit* in CM and SM repectively. In contrast, SM was strongly associated with D816 *c-kit* mutation in group 2, 87% vs. 45% presented 816 mutation in SM and CM, respectively. In patients with SM, presence of 816 *c kit* mutation was associated with adult mastocytosis onset (87% vs. 43% in group 2 and 1, respectively, p<0.001).

**Table 4:** Relationship between phenotype and genotype in patients with mastocytosis according to their age of onset.

WHO classification	Group 1		Group 2		
	wt816	mut 816	wt816	mut 816	
CM (n = 38)	5 (71)	2 (29)	17 (55)	14 (45)	p = 0.788
SM (n = 104)	12 (57)	9 (43)	11 (13)	72 (87)	p<0.001
	p = 0.668		p<0.001		

Data are expressed in number (percentage).

## Discussion

It is currently admitted that pediatrics and adult is mastocytosis exhibit different clinical and genotypic features[2]. To our knowledge, however, only one retrospective study has compared phenotypic characteristics of mastocytosis according to the age of onset [16]. The authors reported more often significant clinical improvement and more frequent gastrointestinal symptoms in childhood onset patients. However, patient's age was unknown, the search for systemic involvement was not investigated in all cases and a long-term follow up was available only for a subgroup of patients.

In the present report, we have demonstrated that although exhibiting genotypic differences, clinical features of adult patients with mastocytosis were similar regardless their adult or pediatric onset.

Overall the percentage of SM was 73% and rates of SM did not differ according to the age of onset. In previous reports, SM was reported to be more frequent in adult mastocytosis patients than in children with mastocytosis, regardless of their outcome. Worobec et al. reported 65 patients with mastocytosis; 90% of the 55 adults studied presented SM, whereas only 30% of the 10 children studied presented SM [7]. In agreement with those findings, previous reports have also shown that bone marrow involvement was significantly more frequent in adult (45%–90%)[17] [18] than in pediatric (18%) [19] patients. These discrepancies with the results of our study might be explained by the fact that pediatric patients with systemic involvement may not resolve at puberty and therefore may not differ from mastocytosis beginning at adult's age. Alternatively, adult patients with mastocytosis related to pediatric onset may have evolved from a cutaneous to a systemic disease because of the longer course of the disease. However, this hypothesis is unlikely since tryptase levels, a marker of mast cell burden, was significantly lower in the group 1.

Our work elucidated that *c-kit* genotype differed with the age of mastocytosis onset. D816X mutation in exon 17 of *c-kit* was more frequent in patients with adult onset mastocytosis than in those with childhood onset. No large previous study had compared *c-kit* genotype in mastocytosis according to age of onset. However, several studies compared *c-kit* genotype in adults and children patients with mastocytosis. Most of the studies reported a higher prevalence of D816X *c-kit* mutations in adults than in children with mastocytosis. Longley et al. reported that all of 10 adult patients (with adult onset) with cutaneous or systemic mastocytosis presented D816 mutation on skin biopsy, whereas only 13% of the 15 children with mastocytosis age of onset between 0 and 12 years had the mutation [9]. In another study, 816 mutation was present in skin for 6 out of 6 adults patients with adult onset (5 patients with SM and 1 with CM), and none of the 11 children patients with CM[10]. A recent study reported 93% of D816V *c-kit* mutation in bone marrow of 113 adult patients with systemic mastocytosis, however, no data on skin biopsy and age of disease's onset was available in this study [20]. Overall these studies suggest that 816 *c-kit* mutation prevalence is high in adult patients and low in pediatrics patients. However, Yanagihori et al reported D816X *c-kit* mutation for 11 out of 13 adults with CM in whom disease started during infancy [12]. In line with this report, in our study we found that 816 mutations was higher (40%) than expected in the population of adults patients with pediatric onset. These findings strongly suggest that 816 mutations in pediatric patients may be associated to a higher probability of persistence at adult age.

Here, non-816 *c-kit* mutations were rarely found in adults' onset patients (2%), whereas it represented 25% of patients with age of onset below 5 years old and 0% of patients with an age of onset between 5 and 15 years. We studied exons 17 and 8 through 13 but not the whole *c-kit* gene because a previous study of our group targeted the entire gene in 50 children and did not find any mutation in other exons of *c-kit* gene (unpublished personal data). Three patients presented Del419D in exon 8 of *c-kit*, 2 patients had a disease onset before the age of 5 and 1 after 15, all presented SM including one with gastrointestinal involvement. This mutation was recently reported in a patient that presented familial gastrointestinal stromal tumor and mastocytosis [21]. In the case reported here, the mutation was not found in peripheral blood mononuclear cells, ruling out a germinal mutation. One of our patients with nonfamilial CM from group 1A presented a K509I mutation in exon 9. Another recent study reported K509I mutation in a case of familial mastocytosis [22]. An adult onset patient with CM/ISM presented V560G mutation in exon11, which has previously been reported in 2 adults, 1 CM and 1 SM [10]. Taken together, these results suggest that in pediatric patients below the age of 15 other mutations than the classical 816 of the *c-kit* gene may occur. Therefore, pathogenesis of mastocytosis clearly differs according to age's onset.

Prospective studies in pediatric population is currently performed to determine whether frequency of these unusual mutations might be a pronostic indicator.

In conclusion, despite similar clinical presentation, the high frequency of non mutated *c-kit* gene in case of childhood's onset contrasted with a high frequency of 816 mutation in mastocytosis cases starting at the adult's age. These findings may have important therapeutic implications when targeted therapy against kinase activity of c-kit is considered.

## Methods

### Patients

Adult patients (>18 years old) with mastocytosis were recruited prospectively through the French mastocytosis network (AFIRMM « French Association for the Initiatives of Research on Mastocyte and Mastocytosis», protocol “Physiopathological and clinical study of mastocytosis in adult patients”) to compare phenotypic and genotypic features of patients according to their age of onset. Data collection was conducted in 15 medical centers between January 2000 and December 2004.

Patients were included after written informed consent was obtained. The study was approved by the ethical committee of Pitie-Salpetrière university hospital (Paris, France) and data were computerized in accordance with the French Commission *Informatique et Libertés*. All patients had a histologically proven mastocytosis. In all cases skin biopsies and bone marrow biopsies/or aspiration were performed. The diagnosis of cutaneous involvement by mastocytosis was based on the association of both clinical symptoms compatible with cutaneous mastocytosis and the presence of a mast cells multifocal accumulation in skin biopsy according to usual histopathological criteria[13], [14]. In most of the cases TMEP doesn't show a significant increase in mast cells and the diagnosis was made in association with clinical datas. However, some subtle features can be useful in the histological diagnosis such as a slight increased number of fusiform and loosely arranged mast cells situated around dilated superficial capillaries. Immunohistochemistry with anti-CD25 was performed on thirty mastocytosis skin samples out of this series, including all various forms of cutaneous mastocytosis, such as urticaria pigmentosa, TMEP and systemic mastocytosis with skin involvement. All cases were negative.

Bone marrow involvement was defined by bone marrow aspiration and/or biopsy with a dense focal or diffuse mast cell infiltrate with spindle-shaped cells [15]. However, in several cases, foci of spindle mast cells located mainly around blood vessels or bone trabeculae were observed which allowed the bone marrow to be classified as involved. Mast cells expressed CD117 and tryptase but in our hands, were rarely stained with CD25 antibody, but were usually closely associated with foci of small B and T-cells.

Liver and/or gastrointestinal involvements were histologically proven. The presence of mast cells within the sinusoids defined liver involvement and the presence of a dense mast cells infiltrate in lamina propria defined gastrointestinal involvement [15]. Clinical, biological and histological data were recorded through a Clinical Records Form (CRF). The following criteria were collected: age at data collection, age of onset of cutaneous involvement based on available written medical reports from a pediatrician or patient's parents, age at diagnosis, gender, cutaneous clinical form (mastocytoma, urticaria pigmentosa, telangiectasia macularis eruptiva perstans (TMEP)), flush, fatigue, anaphylactoid shock, abdominal pain, diarrhea, presence of SM and date of onset, hepatomegaly, splenomegaly, adenomegaly, bone pain, osteoporosis (defined as T score <−2), radiographic skeletal lesion, the presence of bone marrow involvement on bone marrow aspiration and/or biopsy, gastrointestinal, liver involvement, blood cell count, liver enzymes and tryptase serum levels. Bone involvement included osteoporosis and/or compatible radiological bone lesions. Patients were classified according to WHO classification [1], in CM, indolent systemic mastocytosis (ISM), smouldering systemic matocytosis (SSM), aggressive systemic mastocytosis (ASM) or systemic mastocytosis with an associated haematological clonal non-mast cell lineage disease (SM-AHNMD). In cases in which bone marrow aspirate and/or biopsy did not disclose mast cell involvement and that no other histologically organ involvement was proven (liver, gastrointestinal tract), patients were considered to have CM. Group 1 included patients with mastocytosis age of onset ≤15 years old (because this corresponds to the cut off between pediatrics and adult's population in the French health system). This group was divided in group 1A that included patients with age of onset ≤5 years old and group 1B >5 and ≤15 years old. Group 2 included patients with mastocytosis age of onset >15 years old.

### Mutation screening

#### Skin samples

A skin biopsy of 3 to 4 mm on mastocytosis cutaneous lesions was performed for each patient and directly incubated in RNA later (Qiagen) by the dermatologist in charge of the patient before sending it for centralization to the reference laboratory. Skin biopsy was systematically performed at inclusion in this study, therefore at various times from initial mastocytosis diagnosis. We previously assessed that nucleic acids in biopsies were stable for one week at room temperature (data not shown). Samples were stored at −80°C in such buffer upon arrival.

#### RNA preparation and *c-kit* D816V mutation detection

Total RNA was extracted from skin biopsies using the Rneasy mini kit (Qiagen). Complementary DNA was synthesised by using random hexamers and oligo dT as oligonucleotide primer from 200 ng total RNA using the stratascript first-strand synthesis system (Stratagene) in a total volume of 50 µl as recommended by the manufacturer. Then, 2.5 µl of cDNA was introduced in each polymerase chain reaction (PCR).

*c-kit* gene was amplified by PCR using HotStartTaq™ DNA polymerase (Qiagen S.A. France). A total of 40 cycles was performed using either the 9700 or 2700 Gene Amp PCR Systems (Applied biosystems) at 94°C for 30 sec, 57°C for 30 sec and 72°C for 45 sec.

c-Kit coding sequences were amplified from complementary DNA with the PCR by using primer pairs indicated in table 1. For the specific detection of the mutation at the 816 position, we used the primer pairs (2295s & 2661r) indicated in table 5.

**Table 5:** Primer positions are indicated from the c-Kit sequence published through the NCBI accession number X06182.

Name of the PCR primers	Nucleotide Sequence	Localisation (bp)	PCR fragments	Exons amplified
1197s	CCTAGTGTCCAATTCTGACG	1197 to 1216	PCRK3	8 to 13
2100r	GCTTCTGCATGATCTTCCTGC	2100 to 2121		

Name of the sequencing primers	Nucleotide Sequence	Localisation (bp)	PCR fragments	Exons sequenced
1222s	GCTGCCATAGCATTTAATGT	1222 to 1241	PCRK3	8 to 10
1678r	CCTTCCACTGTACTTCAT	1679 to 1696		
1595s	TGCTGATTGGTTTCGTAATCG	1595 to 1615		11 to 13
2029r	CACCATAGCAACAATATTCTG	2030 to 2050		

Name of the ex17 primers	Nucleotide Sequence	localisation (bp)
2295s & 2295sF	GGATGACGAGTTGGCCCTAGA	2295 to 2315
2661r & 2661rF	GTAGAAACTTAGATCGACCGGCA	2639 to 2661
2647r (sequencing)	CGACCGGCATTCCAGGATAG	2628 à 2647

Nested ex17 primers and sequencing	Nucleotide Sequence	localisation (bp)
2341s	TACCAGGTGGCAAAGGGCATG	2341 to 2362
2600r	CTTCCTAAAGAGAACAGCTCC	2600 à 2621

Direct amplimer sequencing was carried out after PCR products were purified with the multiscreen HTS MNSV030050 purification system, (Millipore, Guyancourt France). They were directly sequenced with Big dye terminator V 1.1 (Applied biosystems) on an ABI Prism 3130 sequencer (Applied biosystems) and analysed with the Seqscape software (Applied biosystems) using 2295s & 2647r sequencing primers described in table 5.

#### Confirmation of D816 mutation detection

D^816^V mutation was also confirmed by restriction digest analysis with BsmA1 and Ple1 restriction enzymes, which detect wild type and mutated form, respectively. Purified fluorescent primers (2295sF & 2661rF; see table 1) were used for PCR reactions. Size of restriction digest fragments (201 for Bsma1 fragment and 179 and 187 for Ple1 fragment) were directly determined on a 16 capillary sequencer (ABI Prism 3130 sequencer) with the GeneMapper software (Applied biosystems) by comparison with size of the Genescan rox 500 markers (Applied biosystems).

#### Confirmation of the lack of 816 mutation

In the case of detection of a WT sequence, an independent PCR was performed on cDNA. Same conditions as described above were used for the PCR reaction, except that 30 cycles were used. Then, PCR products were digested by BsmaI enzyme in order to increase a putative mutated signal. The Bsma1 digested products were then amplified in a 25 cycles nested-PCR reaction using 2341s and 2600r primer pairs (see table 5). Same conditions of temperature and time as above were applied for this reaction. Purified nested PCR products were then sequenced with 2341s and 2600r primer pairs as described above and analysed with the Seqscape software.

#### Exons 8 through 13 *c-kit* coding regions were sequenced for WT^816^ patients

A PCR, using the same conditions, was performed on cDNA except that the c-Kit coding region was separated in five different fragments (PCRK1 to K5) for PCR reaction described in table 5. Each PCR fragment was then sequenced as described above using sequencing primer described in table 5.

### Statistical analysis

Statistical analyses were performed using SAS version 9.1 and Excel.

Quantitative variables were summarized using the following descriptive statistics: number of observed and missing data, mean, standard deviation, median, minimum and maximum. Absolute and relative frequency distributions were provided for qualitative variables. We used Student' s T test for continuous variables with limited effectives, and the Wilcoxon test for the nonparametric values. We confronted the values. For the discontinuous values, we used the chi2 test or Fisher's exact test. Statistical significance was defined as p value<0.05.
